# Delayed presentation of shock due to retroperitoneal hemorrhage following a fall

**DOI:** 10.4103/0974-2700.50753

**Published:** 2009

**Authors:** Nader N N Naguib

**Affiliations:** Department of General Surgery, Ain Shams University, Egypt

**Keywords:** Blunt abdominal trauma, retroperitoneal hemorrhage, and Shock

## Abstract

During trauma the abdomen is one region which cannot be ignored. Due to its Complex anatomy it is very important that all the areas in the abdomen be examined both clinically and radiologicaly to rule out any abdominal bleeding as a cause of Hemorrhagic Shock Following Trauma. Our case justifies the above.

## INTRODUCTION

We present a case with unclear history that turned out to have an abdominal cause for his state of shock

## CASE REPORT

A 53-year-old well-built man slipped on the floor and landed on his back. He continued to suffer from sharp abdominal pains gradually increasing in severity for the following few days. The pain was localized in the epigastrium, left hypochondrium and the back. As he overlooked his fall, his local doctor attributed these pains to gastritis.

On the fourth day following his trauma, he presented to the Casualty Unit at Ain Shams University Specialized Hospital with shock and abdominal pain. He could not give relevant history as his level of consciousness continued to deteriorate. None of his family members who were accompanying him knew about his trauma.

His systolic blood pressure was 50–60 mmHg, and his heart rate was 130 bpm. Inspection showed an abdominal mass. The abdominal wall was tender and rigid on examination. The mass was fixed and not pulsating.

Intensive resuscitation measures started immediately. Blood was taken for cross match and investigations. The decision of exploration laparotomy was made. The patient was temporary resuscitated, and on the way to the operating room, he had a rapid CT examination (without contrast) to rule out a ruptured aneurysm due to the lack of proper history. It was difficult to maintain his vital signs that started to deteriorate again; so he was rushed to theatre.

**Figure 1:**
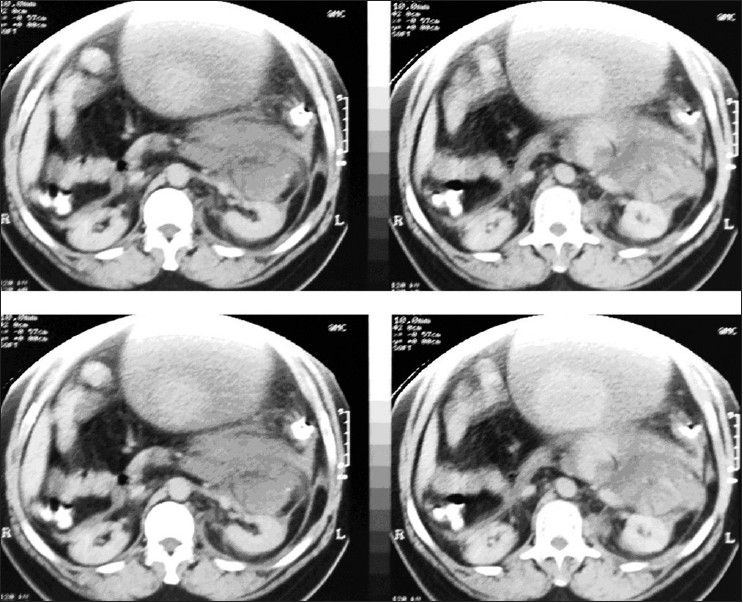
CT scan shows huge multi-locular blood cyst filling most of the abdominal cavity

**Figure 2:**
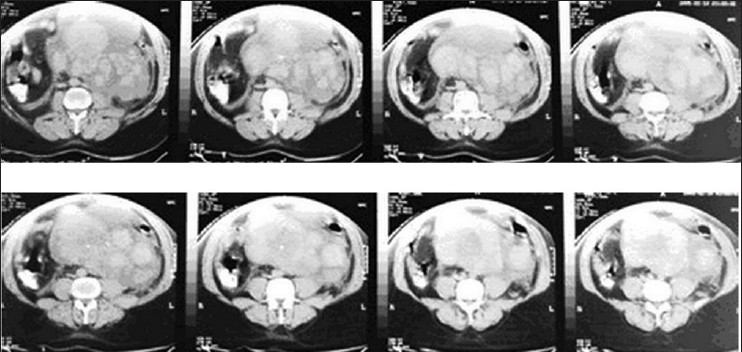
CT scan; multiple cuts show the extent of the blood cyst at different levels

The CT scan showed a huge multi-locular abdominal cyst with blood-dense fluid inside. The origin of the cyst was not clear due to its size. There was a tiny calcified structure floating in cyst cavity. Certainly, the possibility of a leaking aortic aneurysm was excluded. There was no other abnormal finding [Figures 1 and 2].

At the laparotomy, a huge dark blue cyst was filling most of the abdominal cavity and pushing the entire bowel to a small compartment. Opening the cyst revealed dark blood clots and de-fibrinated blood. The cyst was multi-locular as shown on the CT scan. The blood clots were removed and the loculi were traced down to the retroperitoneal space. The retroperitoneal space was full of blood clots of unidentified origin. There was no active bleeding. A small branched atheroma taking the shape of a blood vessel at its bifurcation was retrieved from the cavity of the blood cyst. Its diameter was about 4 mm. It denoted the previous rupture of a retroperitoneal blood vessel at its bifurcation (a lumbar artery). This represented the calcified structure identified at the CT scan.

The patient by this time received 15 units of blood and 12 units of fresh frozen plasma. As a consequence and due to the large size of retroperitoneal hematoma and the fact that there was no active bleeding or identifiable bleeder and the patient started to show signs of coagulopathy, the decision was not to interrupt the retroperitoneal hematoma. The cyst pseudo-wall, which actually was derived from the retroperitoneal wall, was closed by continuous vicryl suture to tamponade the remaining hematoma.

The patient was transferred to the ICU. He stayed there for 3 days and was transferred to the ward. He was discharged 10 days after his laparotomy.

One month later, a follow-up CT scan showed re-accumulation of some blood clots and mild left hydroureter and hydronephrosis. The patient was asymptomatic, but to avoid further pressure on his left kidney, he underwent an elective laparotomy. A smaller multi-locular blood cyst was identified, opened and evacuated. More blood clots were removed and the cyst wall was left open for drainage. The abdomen was closed with two drains inside.

The patient went home on the fifth postoperative day. Following his second laparotomy, he developed a wound infection. Some skin stitches were removed to help the pus draining. Six months follow up by abdominal ultrasound revealed no back pressure on the left kidney and absence of abdominal cysts or masses.

## DISCUSSION

Despite improvements in trauma care, uncontrolled bleeding contributes to 30–40% of trauma-related deaths and is the leading cause of potentially preventable early in-hospital deaths.[1] The presence of the lethal triad of hypothermia, coagulopathy and acidosis represents a major risk for deterioration and adverse outcome of polytrauma patients.[2] Resuscitation of the trauma patient with uncontrolled bleeding requires the early identification of potential bleeding sources followed by prompt action to minimize blood loss, to restore tissue perfusion, and to achieve hemodynamic stability.[1] The clinical symptoms of shock are the three windows to the microcirculation, which can be assessed in terms of inadequate organ perfusion: level of conscious (cerebral perfusion), pulse (peripheral perfusion) and urine output (renal perfusion). These must differentiate whether a patient is hemodynamically normal or just apparently hemodynamically stable. Based on the response to initial resuscitation, the later group of patients is defined as either transient responders or nonresponders and is likely to require a surgical intervention for hemorrhage control.[2]

The problem may be “compensated shock,” in which cellular perfusion lags behind gross physiologic parameters. Not all trauma patients with tissue hypoperfusion as the result of massive hemorrhage arrive at the emergency department with signs of shock.[3]

There is no objective data describing the relationship between the risk of bleeding and the mechanism of injury. Decreasing serial hematocrit measurements may reflect continued bleeding, but patients with significant bleeding may maintain their serial hematocrit.[1] Clinical studies have demonstrated that lactate levels and base deficit represent highly sensitive parameters for recognition of “hidden shock” in traumatic hemorrhage.[2]

The lack of a specific diagnosis should not delay resuscitation when hemorrhage is suggested by history, physical examination, or laboratory findings.[3]

Nevertheless, the delayed presentation of an abdominal bleeding in a victim of a fall is rare. Wheaton and Tsalamandris presented a 17-year-old boy with delayed manifestation of abdominal bleeding 24 hours after a fall.[4] In this case, the patient presented on the fourth day after his fall.

Hematoma and bleeding in the retroperitoneal space are observed in 44% of patients with blunt abdominal trauma. Injuries missed during the in-hospital secondary survey are responsible for a mortality- rate up to 50% due to delayed treatment, while on the contrary, an early diagnosis does not carry high incidence of severe complications.[5]

Evaluating patients who have sustained blunt abdominal trauma remains one of the most challenging aspects of acute trauma care. Physical examination findings are notoriously unreliable as large amounts of blood can accumulate in the peritoneal and pelvic cavities without any significant or early changes in physical examination findings.[6]

For patients with blunt abdominal trauma, it might be worthy to image the abdomen and pelvis to rule out a concomitant occult abdominal injury. According to the European guidelines by the Multidisciplinary Task Force for Advanced Bleeding Care, trauma patients presenting with hemorrhagic shock and an unidentified source of bleeding should undergo immediate further assessment.[7] Although it produces highly sensitive findings in well-trained hands, FAST, unlike CT, is not specific to the site of origin and extent of injury.[8] CT scan often provides detailed diagnosis in cases with misleading symptoms and thus determines the need for operative intervention. The best CT imaging requires both oral and IV contrast.[9] Stuhlfaut and colleagues concluded that acquisition of initial CT scans without oral contrast material helps us to meet both safety (definite risk of aspiration, and the potential delay in patient care) and efficiency without sacrificing diagnostic accuracy.[10] The decision to operate upon the patient in the reported case study was made because the patient was in shock. The CT scan we conducted is a controversial issue. It was done without contrast and without really having the appropriate time for a detailed radiological study. But because of the absence of a detailed history of his trauma, we decided to exclude a ruptured aneurysm or other extra- abdominal cause of bleeding.

For patients with major injuries isolated to the abdomen requiring emergency laparotomy, the probability of death shows a relationship to both the extent of hypotension and the length of time in the emergency department for those who were there for 90 min or less.[11] Hill and colleagues observed a significant decrease in mortality from shock by introducing an educational program on trauma and by establishing a 60-min emergency department time limit for patients in a state of hemorrhagic shock.[12] In our case study, it took less than 60 min to transfer this patient to the operating room including the time spared for the CT scan.

Retroperitoneal hemorrhage caused by blunt injuries still remains a challenge for the surgeons as there is always a risk of turning it into unstoppable bleeding. Muftuoglu *et al.*, emphasized that a treatment strategy must be determined by the surgeon, considering the ages, type of injury, accompanying diseases, additional organ injuries and especially the hemodynamic stabilities of the patients. Perforated hollow viscous and hemodynamic parameters that cannot be stabilized are the indications of operation.[13] In 1983, Stone and colleagues described the techniques of abbreviated laparotomy, packing to control hemorrhage and of deferred definitive surgical repair until coagulation had been established.[14] Since then, a number of authors have described the beneficial results of this concept, which is now called “damage control.”

Damage control was done in this case, and the peritoneum was sutured to prevent the spread of the hematoma. The patient was transferred to ICU, and later he had another elective surgery to deal with the back pressure on the kidney.

Retroperitoneal bleeding secondary to interruption of lumbar and pelvic arteries are the most common cause of hemorrhagic shock from vertical deceleration injuries.[15] Siablis and colleagues reported a case survived after a fall and had a relatively small retroperitoneal hematoma detected during urgent splenectomy. It was underestimated; 2 weeks later, the underlying laceration of the lumbar artery led to the formation of a pseudo-aneurysm, which then ruptured causing a large retroperitoneal hematoma and gradual complete femoral nerve palsy. Traumatic rupture of a lumbar artery is a rare complication of a blunt abdominal trauma that can lead to a potentially massive retroperitoneal hemorrhage and shock, or to subsequent pseudo-aneurysm formation and delayed retroperitoneal hematoma.[16] In this case, the exact origin of bleeding was not identified as there was no active bleeding. It was suggested that the cause of hemorrhage was a ruptured lumbar artery due to the floating calcified atheroma retrieved from the blood cyst. The atheromatic surgical finding was shaped as a blood vessel with a diameter of 4-5 mm, which indicated the lining of a lumbar artery.

A mortality rate between 18% and 60% is observed in the patients with retroperitoneal hematoma. The main cause of death is hemorrhagic shock. As a surgical strategy, exploring a retroperitoneal hematoma should be the last resort for the surgeon if only there is severe active retroperitoneal bleeding as opening of this closed system imposes “a chimney effect” and when the pressure on the bleeding tissues is taken-off; the bleeding may become even more severe and lethal. Muftuoglu *et al.*, reported in their study that more than half of the patients with a retroperitoneal hematoma died postoperatively due to the decision for retroperitoneal exploration.[13] Many collateral vessels supply any particular region of the retroperitoneum. This can explain why once a bleeding artery is under control, collateral supply to the same region could result in new bleeding. This is one of the major reasons why the surgeons avoid exploration of the retroperitoneum in the setting of trauma. Also lumbar arteries in particular are difficult to control operatively.[17] As damage control surgery was applied in our case, we did not risk further exploration of the hematoma. It is more important to save the patient than to control the hematoma.

Resuscitation is an ongoing process. Throughout the resuscitation and assessment phase the care providers need to keep an eye on the big picture – what are the patient's injuries and what is the highest priority? Transfer to definitive care, especially the operating room, should not be delayed by lower priority concerns.[18]

Despite the lack of definitive scientific evidence, numerous research studies and requests for funding are based on achieving the “Golden Hour” for all trauma patients.[19] In blunt polytrauma patients, this early phase of the golden hour is not restricted to management within the first 60 min after injury only, but can be safely extended to the first few hours after trauma.[20]

The first 24 hours of resuscitation is strongly associated with increased morbidity and mortality. There is a post-traumatic therapeutic window of 24 h (the silver day), which if used efficaciously may dramatically influence the outcome of trauma patients.[21]

The established time-dependant management phases for trauma patients in the first 24 h comprise the following:

1. Primary survey with baseline diagnostics and immediate life-saving procedures according to the ABC algorithm of the ATLS protocol.
2. Damage control surgery in patients who are not responsive to the initial measures of resuscitation.
3. Secondary survey, which can only be started in the hemodynamically stable patients including a head to toe examination and further radiologic work up.
4. Delayed primary surgery; surgical interventions which are not immediately required for resolving life threatening conditions are performed after further evaluation of the stabilized patients in the secondary survey.[2]

Tertiary survey in the first 24 h can still miss injuries. Vigilance and careful re-examination is required to minimize the impact of these injuries and to effect treatment as early as possible.[22]

End points of resuscitation are as follows:

1. Stable hemodynamics without need for vasoactive or inotropic stimulation
2. No hypoxemia or hypercapnia
3. Serum lactate < 2 mmol/l
4. Normal coagulation
5. Normothermia
6. Urine output >1 ml/kg/h[23]

## CONCLUSION

Bleeding in the retroperitoneal tissue can present late following blunt abdominal trauma. It can be massive and lead to hemorrhagic shock. It should be among the differential diagnosis in patients with clinical diagnosis of hemorrhagic shock even in absence of evident history of trauma as rapid and prompt management decrease the mortality rate. Damage control surgery should be employed for patients presenting with deep hemorrhagic shock and coagulopathy. Close observation and follow up is required as further management is likely required. Further studies are required to investigate the need of follow up imaging for trauma patients to detect the delayed complications at an early stage.
